# Academic Performance of Children With Sickle Cell Disease in the United States: A Meta-Analysis

**DOI:** 10.3389/fneur.2021.786065

**Published:** 2021-12-13

**Authors:** Andrew M. Heitzer, Latacha Hamilton, Claire Stafford, Jeffrey Gossett, Lara Ouellette, Ana Trpchevska, Allison A. King, Guolian Kang, Jane S. Hankins

**Affiliations:** ^1^Psychology, St. Jude Children's Research Hospital, Memphis, TN, United States; ^2^School Program, St. Jude Children's Research Hospital, Memphis, TN, United States; ^3^Psychology, Nova Southeastern College of Psychology, Fort Lauderdale, FL, United States; ^4^Biostatistics, St. Jude Children's Research Hospital, Memphis, TN, United States; ^5^Health Sciences Resource Center, Texas Medical Center Library, Houston, TX, United States; ^6^Program in Occupational Therapy and Departments of Pediatrics and Medicine, Washington University, St. Louis, MO, United States; ^7^Hematology, St. Jude Children's Research Hospital, Memphis, TN, United States

**Keywords:** sickle cell, anemia, academic performance, neurocognitive, education, stroke, silent infarct, grade retention

## Abstract

**Background:** Students with sickle cell disease are at risk for poor academic performance due to the combined and/or interactive effects of environmental, psychosocial, and disease-specific factors. Poor academic performance has significant social and health consequences.

**Objective:** To study academic achievement and attainment in children with sickle cell disease in the United States.

**Design:** Medline, Embase, SCOPUS, CINAHL, ERIC, and PsycINFO were searched for peer-reviewed articles. Studies of children (ages 5–18) diagnosed with sickle cell disease of any genotype reporting academic achievement (standardized tests of reading, math, and spelling) or attainment (grade retention or special education) outcomes were included. Outcomes were analyzed using a random effects model. Achievement scores were compared to within study controls or normative expectations. Prevalence of special education services was compared to national (United States) estimates for Black students. Age at assessment and overall IQ were evaluated separately for association with reading and mathematics scores. Subgroup analyses of reading and math scores were analyzed by cerebral infarct status (no cerebrovascular accident, silent infarct, stroke).

**Results:** There were 44 eligible studies. Students with sickle cell disease scored 0.70, 0.87, and 0.80 (*p* < 0.001) SD below normative expectations on measures of reading, mathematics, and spelling, respectively. Compared to unaffected sibling and/or healthy controls (*k* = 8, *n* = 508), reading and math scores were 0.40 (*p* = 0.017) and 0.36 (*p* = 0.033) SD below expectations. Intellectual functioning explained 97.3 and 85.8% of the variance in reading and mathematics performance, respectively (*p* < 0.001). Subgroup analyses revealed significant differences in reading (*p* = 0.034) and mathematics (*p* < 0.001) based on infarct status, with lower performance associated with presence of a silent infarct or stroke.

**Conclusion:** Students with sickle cell disease demonstrate notable academic difficulties and are at high risk for grade retainment. Development of academic interventions and increased access to school support services are needed for this vulnerable population.

**Systematic Review Registration:** https://www.crd.york.ac.uk/prospero/display_record.php?ID=CRD42020179062.

## Introduction

### Rationale

Students with sickle cell disease (SCD) are at risk for poor academic performance (1, 2). These academic difficulties have a substantial effect on quality of life and potential for future income. Approximately 30% of students with SCD do not graduate high school (3). These students with limited academic attainment are at much higher risk for unemployment (4) and have a significantly greater frequency of acute care hospitalizations (5). Thus, it is important to understand the extent of these academic difficulties in the SCD population to potentially intervene and prevent future academic failure.

SCD is one of the most common genetic hemoglobin disorders, occurring in approximately one in 400–500 African Americans in the United States (6). SCD is caused by a single point mutation that generates the abnormal hemoglobin S (HbS). The HbS altered behavior leads to red blood cell adhesion and vaso-occlusion, blocking oxygen and blood flow to vital organs and bones. This blockage results in episodic pain and may lead to cerebrovascular injuries, including silent cerebral infarcts (SCI) and overt strokes (7). Students with SCD often require frequent hospital admissions due to pain crises and other acute complications (8) and display high levels of fatigue (9).

An extensive literature has demonstrated the combined and/or interactive effects of environmental (10, 11), psychosocial (8, 12), and disease-specific (13, 14) factors on cognitive functioning in SCD, yet much less is known regarding academic performance. Academic and cognitive performance share many of the same risk factors (e.g., cerebrovascular injury, chronic anemia, socioeconomic status); however, they are unique constructs. In the general population, environmental factors account for a greater amount of variance in academic performance than cognitive ability (15, 16). This is particularly relevant for students with SCD, as they are more likely to be raised in single-parent families, live in low-income resource-poor neighborhoods, and attend financially disadvantaged school systems (17). Students with SCD miss an average of 20–40 school days per year due to acute pain, fatigue, and frequent outpatient visits (18), which are associated with poor academic performance and grade retention (19, 20). Social and behavioral difficulties (21, 22) observed in SCD (e.g., anxiety, depression, limited sleep) may further interfere with functioning in the school environment.

To assess academic performance, it is important to consider both academic knowledge (e.g., test achievement measures) and attainment (e.g., history of retention, graduation rates). Academic achievement skills are necessary for performing well in school but are not sufficient (23). Executive dysfunction (e.g., attention deficit) and hospital visits due to acute pain can interfere with school performance among students with age-appropriate achievement skills. For example, a student may possess the knowledge to perform well on a test or complete a homework assignment, yet they are unable to adequately display their knowledge as they cannot focus throughout the exam or cannot complete the assessment test in the allotted time. This distinction is highly relevant for intervention. Students with executive difficulties are likely to benefit from behavioral therapy or organizational support, whereas those with knowledge deficits require remediation and/or tutoring in subject area content.

There exists a range of federal, state, and local laws, regulations, and systems for special education and related services for children and adolescents with SCD. In brief, special education services are mandated through federal and state law under the Individuals with Disabilities Education Act (IDEA), typically taking the form of an Individualized Education Plan (IEP). If a student does not qualify for an IEP, related services are justified through Section 504 of the Rehabilitation Act of 1973, which prohibits discrimination based on disability within federal and federally assisted programs. The definition of a disability under Section 504 is much broader than in an IEP and often includes children with medical conditions such as SCD with no observed learning/cognitive deficits (24). In practice, the most important distinction between Section 504 and an IEP is that Section 504 is intended to eliminate barriers for students with disabilities whereas an IEP is remedial and often requiring the provision of programs and services (25).

To our knowledge, there have been five meta-analyses that have assessed cognitive deficits in individuals with sickle cell disease (26–30), however; none of these studies included academic achievement measures or academic attainment outcomes. The most recent, and comprehensive, meta-analysis conducted by Prussien et al. (26, 27), demonstrated deficits in cognitive functioning across several domains. They also reported a gradient of severity, with the most severe cognitive deficits observed in individuals with stroke, followed by SCI, and no cerebral vascular injury. Unlike previous meta-analyses, Prussien and colleagues compared SCD patients to normative expectations in addition to sibling or healthy controls. This comparison allowed for further consideration of sociodemographic factors effects on outcomes that are not fully captured when compared to individuals of similar socioeconomic status. Our objective is to quantitatively review academic outcomes in SCD, addressing both academic achievement and attainment, domains which have yet to be reviewed in SCD. We hypothesized that students with SCD would perform below normative expectations and/or controls across measures of academic performance. We further hypothesized that worse academic achievement performance would be associated with increased age, reduced intelligence scores, and presence of a cerebral infarct.

### Objectives

The primary focus of this meta-analytic review is to provide the first comprehensive quantitative analysis of academic achievement and attainment in SCD. Thus, our first objective was to assess reading, arithmetic, and spelling achievement skills as well as rates of grade retention and special education within the school environment in children with SCD. We compared reading, arithmetic and spelling academic outcomes to both normative expectations and sibling or healthy controls. Secondly, we aimed to evaluate if age at assessment and intellectual functioning moderated academic performance. Finally, we examined if academic achievement outcomes differed by cerebral infarct status.

## Methods

To increase transparency and reproducibility, our review complies with the Preferred Reporting Items for Systematic Reviews and Meta-Analyses (PRISMA) reporting guidelines.

### Protocol Registration

Prior to starting the review, we developed our study protocol, which was published in PROSPERO, an international database of prospectively registered systematic reviews, on 5/7/2020, and assigned the identifier: CRD42020179062.

### Eligibility Criteria

Inclusion criteria for study selection were (1) the study concerned individuals with SCD of any genotype; (2) age at academic assessment was between 5 and 18 years of age; (3) academic performance was assessed using standardized tests with reliability and validity statistics; (4) academic attainment was assessed via grade retention or special education services (e.g., Section 504 Plan, Individualized Education Plan); (5) publication was in a peer-reviewed journal as a full manuscript, (6) and publication was in English and concerned students in the United States. Studies were excluded if (1) the average age of the sample was > 18.0 years; (2) only questionnaire-based assessments of academic achievement or grades were reported; (3) and publication involved intervention for cognitive or academic performance. If pre-intervention measures were reported, however, only these were included (not post-intervention) in the meta-analysis.

### Information Sources and Search Strategy

We searched the following electronic bibliographic databases: Medline (Ovid) 1946–2020, Embase (embase.com) 1974–2020, SCOPUS (2004–2020), CINAHL (1937–2020), ERIC 1966–2020 (eric.ed.gov), and PsycINFO (APA) 1967-2020. A medical librarian (L.O.) developed the primary search in Medline, with three main concepts: (1) sickle cell, (2) academic performance and achievement, and (3) children. Each concept was developed using both controlled and natural languages. MeSH terms were identified, and keywords were gathered along with various synonyms. The keywords were searched using the title, abstract, and keyword fields within the Medline OVID database before being translated to other databases. The final Medline search strategy is found in our published protocol in PROSPERO (refer to Supplementary Table 1). Citation management and duplicate removal was accomplished with EndNote (Clarivate Analytics).

### Study Selection

All titles and abstracts of studies were independently screened by two reviewers (A.H. and L.H.) to identify studies meeting the inclusion criteria. Discrepancies were resolved through discussion or through an arbitrator (J.H.). Covidence (www.Covidence.org) (31) was used to screen and review studies.

### Data Extraction and Coding

Data from the final selection of eligible articles were independently extracted by two review authors (A.H. and L.H.) using a standardized template. Study data included: number and age of subjects, sex, disease genotype, cerebral infarct status (SCI, stroke, or none), and type of control subject (healthy, sibling, or none). Academic performance variables included: reading, arithmetic, and spelling performance. Reading performance incorporated measures of basic word reading and reading comprehension. Arithmetic performance was based on measures of arithmetic calculation or applied mathematics. Spelling performance was based on a single subtest in all studies. Composite scores were computed for studies that reported multiple arithmetic or reading subtests based on the weighted average of each subtest. A list of academic achievement measures used is provided in Supplementary Table 1. All performance data were converted to an age-standardized score with a mean of 100 and a standard deviation (SD) of 15.

For special education and grade retention, percentages of SCD and control children receiving any form of special assistance or requiring grade retention were collected. Descriptions of special education assistance by study are provided in the Supplementary Material. Due to extensive variability in how special education was defined and availability of normative data, only IEP status was examined in analyses. Normative data for IEP services (IDEA Part B) and grade retention were extracted from U.S. national datasets. Normative values for IEP services (IDEA Part B) were based on U.S. Department of Education, Office of Special Education Programs, Individuals with Disabilities Education Act (IDEA) database for the average percentage of Black students (3–21 years) receiving services through IDEA Part B from 2000 to 2019 (32).

### Study Quality Assessment

Two review authors (A.H. and L.H.) independently assessed study quality in chosen studies. A version of the National Institutes of Health Quality Assessment Tool for Observational Cohort and Cross-Sectional Studies adapted by Prussien et al. (26) was utilized. Studies received one point for each criterion met, for a total score of 0–6 (higher values indicate higher study quality). Information about quality ratings for included studies are depicted in Supplementary Table 2. Study quality ranged 3–6 (Mean = 4.3, SD = 0.9).

### Statistical Analyses

For studies based on the same sample or a subset of a sample, only the study with the largest available sample was included. Cochran's *Q*-value, τ^2^ and/or the *I*^2^ value were used to quantify heterogeneity. For example, the amount of heterogeneity (i.e., τ^2^), was estimated using the restricted maximum-likelihood estimator. The Q-value was used to test whether differences in studies are due to systemic differences or to chance alone. The *I*^2^ value assesses the percentage of inter-study variability that can be attributed to heterogeneity. Forest plots were used to display study results, effect sizes, measures of heterogeneity and statistical tests.

Reading, mathematics, and spelling outcomes were compared to normative expectations and controls. The normative comparisons were based on comparisons to a normal distribution with a mean of 100 and a standard deviation of 15. For each study and outcome, *z*-scores were constructed by subtracting 100 from each study mean and dividing by the resulting value by the standard deviation of 15. Study standard deviations were transformed by dividing by the normative standard deviation of 15. For studies with a sample of appropriate controls, patients with SCD were compared to controls. The Hedges' g was used as an estimate of the effect size. Standardized differences were calculated by multiplying Hedges' g by SD = 15.

Age at assessment and overall IQ were evaluated separately for association with reading and mathematics outcomes. Age and IQ were entered as linear predictors for reading and math composite scores. The pseudo *R*-squared value was computed as percent reduction in estimated τ^2^ [amount of heterogeneity as estimated based on a random-effects model vs. the amount of (residual) heterogeneity as estimated based on the mixed-effects meta-regression model with predictors].

Pooled estimates of the proportions using services of Special education, 504, and IEP services and grade retention were estimated using random effects models. Heterogeneity of studies was also evaluated. For IEP's, normative data were available, and comparisons were made via odds-ratios. Normative values for IEP services (IDEA Part B) were based on the average percentage of African-American students (3–21 years) receiving services through IDEA Part B from 2000 to 2019.

Subgroup analyses of assessments of reading and math composite scores were analyzed by cerebral infarct status. Within group heterogeneity and between group variation parameters were quantified using mixed-effects meta regression models.

Thus, for each outcome of interest either a random-effects model or mixed-effects (when moderator was present) was fitted to the data for each outcome. The amount of heterogeneity (i.e., τ^2^), was estimated using the restricted maximum-likelihood estimator (33). The *Q*-test for heterogeneity (34) and the *I*^2^ statistic (35) were evaluated. Studentized residuals and Cook's distances were used to identify possible outliers and/or influential studies (36). The regression test (37) was used to evaluate funnel plot asymmetry. The analysis was carried out using R (version 4.1.0) (R Core Team, 2020) and the metafor package (version 3.0.2) (38).

## Results

**Figure 1:**
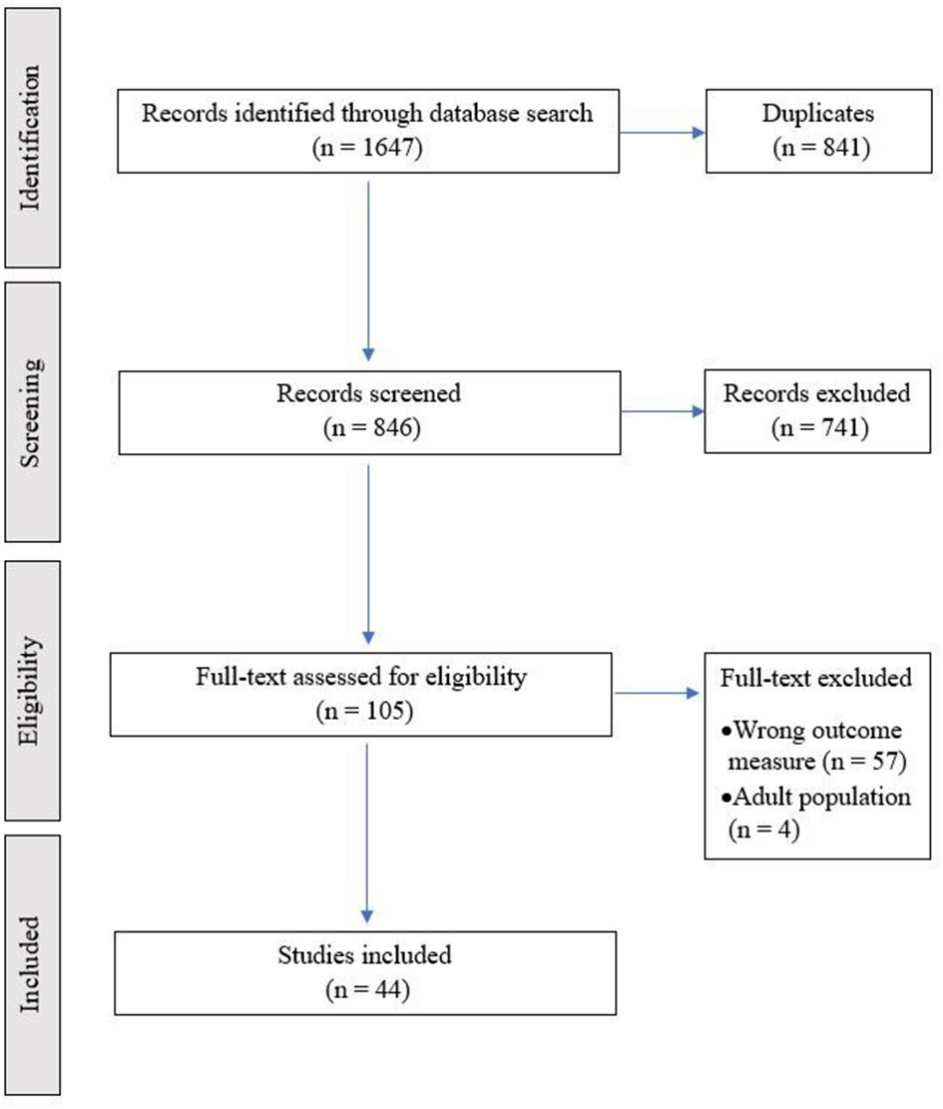
PRISMA flow chart of study. All titles and abstracts of studies were independently screened by two reviewers (A.H. and L.H.) to identify studies meeting the inclusion criteria. Discrepancies were resolved through discussion or through an arbitrator (J.H.). Covidence (www.Covidence.org) was used to screen and review studies.

A total of 846 abstracts were screened for inclusion (see Figure 1). A total of 105 studies met initial screening criteria. Of those, 57 studies were removed for not reporting outcomes of interest (e.g., only measures of cognition or no academic attainment outcomes reported) and 4 studies were excluded for having an adult population (i.e., average age over 18). A final sample of 44 studies (total of 3,971 children with SCD) met criteria (Figure 1). Overlapping samples are displayed in Supplementary Table 2.

Reading, mathematics, and spelling outcomes were assessed via performance measures in 20, 18, and 7 unique studies, respectively (Table 1). Of these, 8 studies assessing reading and math performance included a control group of patients without SCD, and only 4 studies of spelling performance recruited controls.

**Table 1:** Reading, mathematics, and spelling outcomes compared to normative expectations and controls.

	**SCD vs. Normative expectations**						**SCD vs. Controls**					
**Outcome**	***k***	***n***	**ΔSS**	***g***	**SE *g***	***p*-value**	***k***	***n***	**ΔSS**	***g***	**SE *g***	***p*-value**
Reading	20	1,520	−10.6	−0.70	0.08	**<0.001**	8	508	−6.0	−0.40	0.17	**0.017**
Math	18	1,369	−13.1	−0.87	0.10	**<0.001**	8	508	−5.4	−0.36	0.17	**0.033**
Spelling	7	279	−12.0	−0.80	0.17	**<0.001**	–	–	–	–	–	–

*SCD, sickle cell disease; normative expectations, normative values based on sample used to create performance measures; controls, siblings or demographically matched controls (varies by study); k, number of unique studies included in analyses; n, total sample size from all included studies; ΔSS, difference in scores from normative expectations or controls (Mean = 100, SD = 15) calculated by multiplying Hedges' g by SD = 15; g = mean effect size Hedges' g). SE g = standard error for Hedges' g effect size*.

*Reading performance incorporated measures of basic word reading and reading comprehension. Math performance was based on measures of arithmetic calculation or applied mathematics. Spelling performance was based on a single subtest in all studies. Composite scores were computed for studies that reported multiple arithmetic or reading subtests based on the weighted average of each subtest*.

*p-value < 0.05 was in bold*.

Ten studies reported IEP status among 1,210 students with SCD (Table 2). Grade retention was assessed in 16 studies, including 1,803 students with SCD. Only 3 studies reporting IEP status or grade retention included a control group. Participants' ages ranged from 5 to 18 years across all studies.

**Table 2:** Provision of special education services and rates of grade retention in sickle cell disease compared to rates for African Americans nationally.

**Outcome**	***k***	***n***	**Percentage (95% CI)**	**OR (95% CI)**	***p*-value**
Special education	19	1,669	38% (29%, 47%)	**–**	**–**
504 plan	5	808	10% (6%, 15%)	**–**	**–**
Individualized Education Plan (IEP)	10	1,210	32% (22%, 41%)	2.4 (1.5, 3.7)	**<0.001**
Retention	16	1,803	27% (22%, 33%)		

*k, number of unique studies included in analyses; n, total sample size from all included studies; CI, confidence interval; OR, odds ratio for comparison to normative data. Normative values for IEP services (IDEA Part B) were based on the average percentage of Black students (3–21 years) receiving services through IDEA Part B from 2000 to 2019. IEP normative value of 15.6% based on average counts per year from 2000 to 2019: (1,261,827/8,068,662)*.

*p-value < 0.05 was in bold*.

### Reading, Math, and Spelling Performance

Results revealed significant differences in math, reading, and spelling performance compared to age-normative expectations (Table 1). Reading, math, and spelling scores were 10.6 (*g* = −0.70), 13.1 (*g* = −0.87), and 12.0 (*g* = −0.80) points below normative expectations (*p* < 0.001), respectively, consistent with medium to large effect sizes. Results for SCD vs. normative reading, math, and spelling scores were all highly heterogeneous across studies (Tau^2^ = 0.11–0.18, *p* < 0.01). Therefore, pooled results should be interpreted with caution.

**Figure 2:**
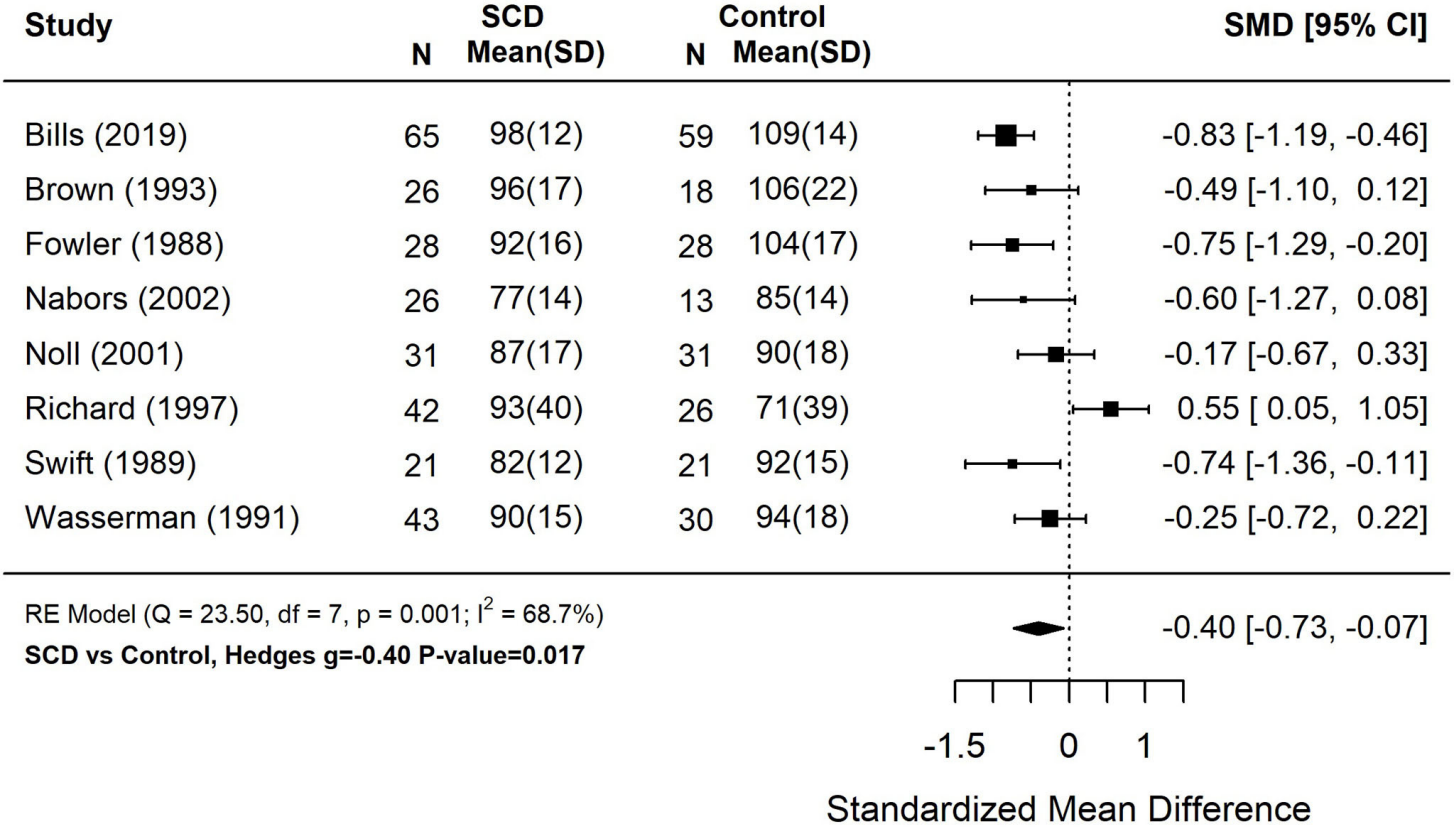
Forest plot for composite reading score among patients with sickle cell disease compared to controls. Reading performance incorporated measures of basic word reading and reading comprehension. Composite scores were computed for studies that reported multiple reading subtests based on the weighted average of each subtest. *N*, the sample size per study subgroup. SD, standard deviation. SMD, standardized mean difference. 95% CI, 95% confidence interval.

**Figure 3:**
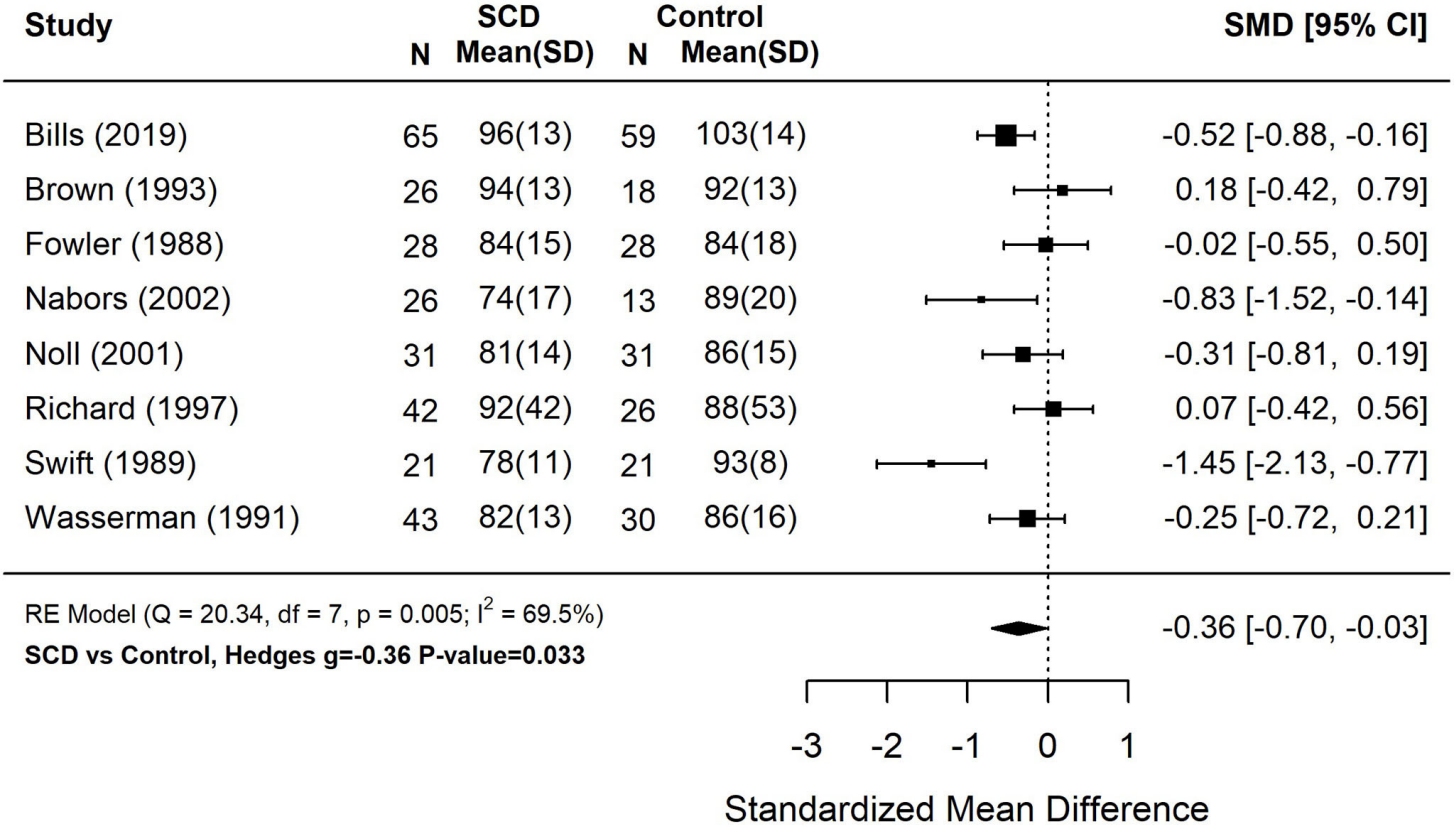
Forest plot for composite math score among patients with sickle cell disease compared to controls. Math performance based on measures of arithmetic calculation or applied mathematics. Composite scores were computed for studies that reported multiple arithmetic subtests based on the weighted average of each subtest. *N*, the sample size per study subgroup. SD, standard deviation. SMD, standardized mean difference. 95% CI, 95% confidence interval.

Reading scores of students with SCD fell 6.0 (*g* = −0.40) points below demographically matched and/or sibling controls without SCD (Figure 2). Performance on measures of mathematics were 5.4 (*g* = −0.36) points below matched controls (Figure 3). These studies also displayed a great amount of heterogeneity in findings (Tau^2^ = 0.15–0.16, *p* < 0.01). Only 4 studies assessing spelling performance recruited a control group, therefore we did not calculate pooled group differences.

### IEP Support and Grade Retention

Based on the 10 studies with IEP data (Table 2), the pooled estimate for percentage of students with SCD having an IEP was 32% (95% CI: 22, 41%). When comparing the portion of students with SCD who have an IEP to a normative value of 15.6%, the estimated pooled odds ratio based on the random-effects model was 2.4 (95% CI: 1.5–3.7, *p* < 0.001). Thus, children with SCD were more than 2 times more likely to receive an IEP than African American students nationally.

Based on 16 studies including rates of grade retention, the pooled estimate was 27% (22, 33%).

### Age and IQ Moderation of Reading and Math Performance

As displayed in Table 3, across 19 studies that measured reading performance, older age was marginally associated with poorer reading performance (Estimate = −0.99, Standard Error = 0.52, *p* = 0.056). Consistently, age was negatively associated with math performance across 17 studies (Estimate = −1.72, Standard Error = 0.83, *p* = 0.037).

**Table 3:** Moderator analyses of reading and mathematics by intellectual functioning and age at assessment.

	**IQ**				**Age**					
**Outcome**	***k***	**Beta**	***p*-value**	***I* ^2^**	***R* ^2^**	***k***	**Beta**	***p*-value**	***I* ^2^**	***R* ^2^**
Reading	13	0.76	**<0.001**	11.22	97.3	19	−0.99	0.056	84.60	18.0
Math	11	0.84	**<0.001**	51.82	85.8	17	−1.72	**0.037**	88.02	21.0

*IQ, intelligence quotient captured from a validated intelligence measure (mean = 100, standard deviation = 15); Age, average age (in years) of the study sample; k, number of unique studies included in the analyses. Reading performance incorporated measures of basic word reading and reading comprehension. Math performance was based on measures of arithmetic calculation or applied mathematics. Spelling performance was based on a single subtest in all studies. Composite scores were computed for studies that reported multiple arithmetic or reading subtests based on the weighted average of each subtest*.

*I^2^ estimates the amount of heterogeneity relative to the total variance. For example, in the model with reading as the outcome and IQ as the moderator, we estimate that 11% of the total variance is due to heterogeneity*.

*R^2^, value is computed as percent reduction in estimated Tau^2^ which is computed using the difference in Tau^2^ estimates from the random effects model vs. the mixed-effects meta-regression model (i.e., with predictor)*.

*p-value < 0.05 was in bold*.

Both reading and mathematics were highly associated with overall IQ across studies (Table 3). After inclusion of IQ as a moderator in analyses, 11.2 and 51.8% of the variability in reading and mathematics outcomes, respectively, can be attributed to the remaining between-study heterogeneity.

### Academic Performance by Infarct Status

**Figure 4:**
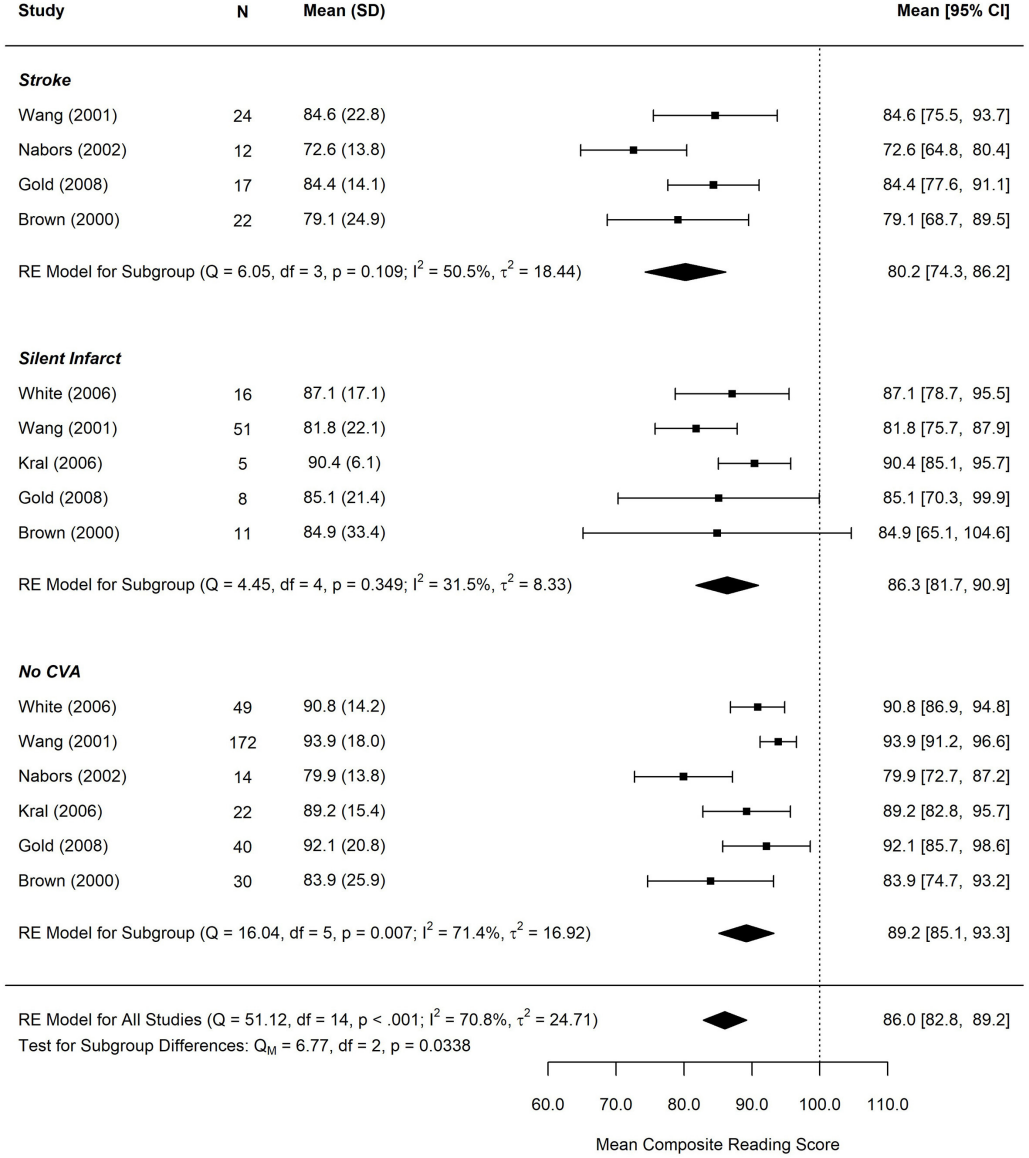
Forest plot for Mean Composite Reading score by infarct status. CVA, cerebrovascular accident. Reading performance incorporated measures of basic word reading and reading comprehension. Composite scores were computed for studies that reported multiple reading subtests based on the weighted average of each subtest. *N*, study subgroup sample size. SD, standard deviation. 95% CI, 95% confidence interval.

**Figure 5:**
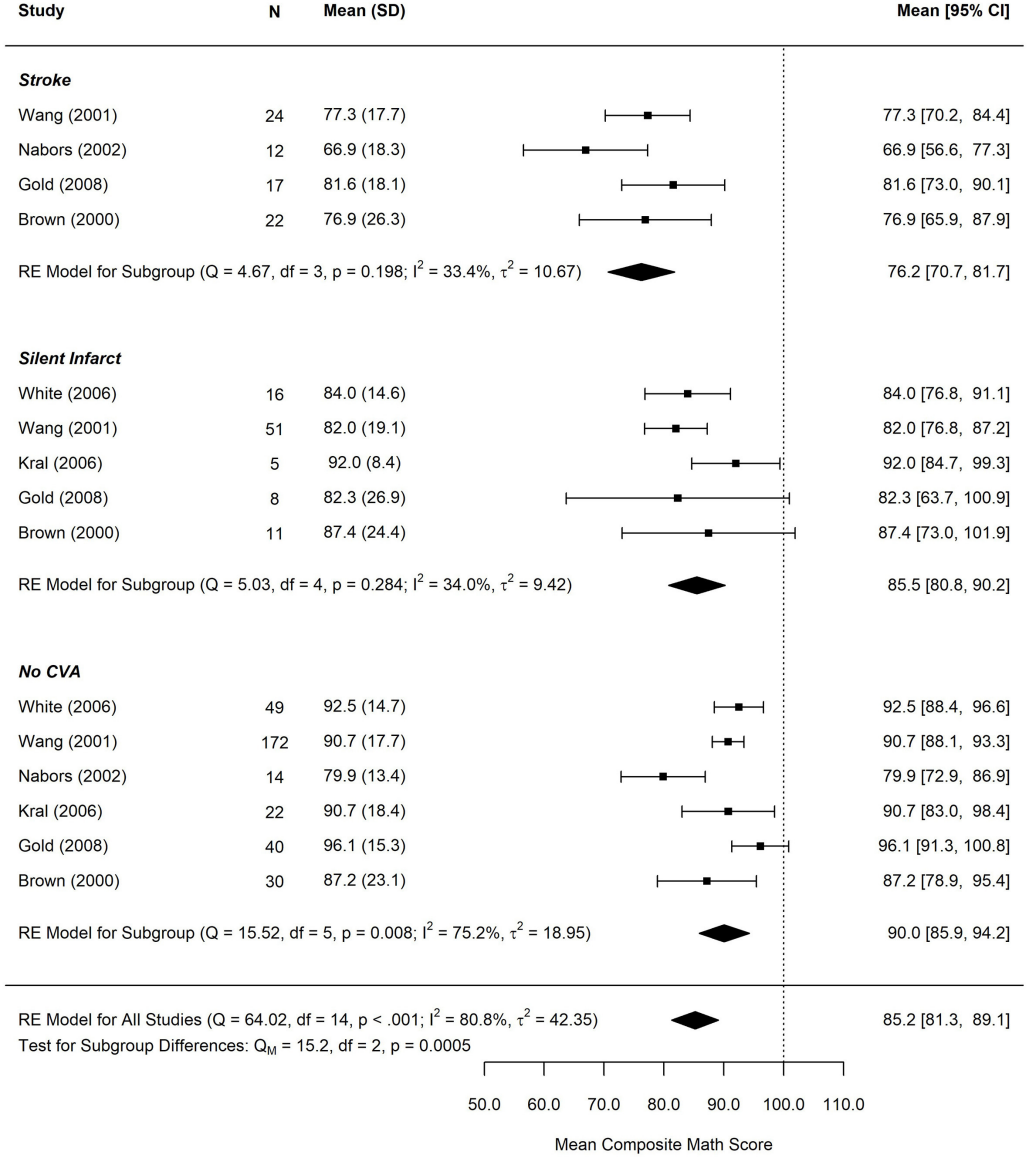
Forest plot for composite math score by infarct status. CVA, cerebrovascular accident. Math performance based on measures of arithmetic calculation or applied mathematics. Composite scores were computed for studies that reported multiple arithmetic subtests based on the weighted average of each subtest. SD, standard deviation. *N*, study subgroup sample size. 95% CI, 95% confidence interval.

Figures 4, 5 display reading and math performance for SCD patients with stroke, silent cerebral infarct, or no cerebrovascular accident (CVA).

The test for subgroup differences in composite reading scores by cerebral infarct status suggests that there was a statistically significant subgroup effect (*p* = 0.034), meaning that cerebral infarct status significantly affects reading performance. However, there was substantial unexplained heterogeneity between the studies within each of the subgroups (no CVA: *I*^2^ = 50.5%, silent cerebral infarct: *I*^2^ = 31.5%, and stroke: I^2^ = 71.4%). The mean reading score was 80.2 (95% CI: 66.7–86.2) in the stroke group, 86.3 (95% CI: 81.7–90.9) in the silent infarct group, and 89.2 (95% CI: 85.1, 93.3) in the group without history of a CVA.

The test for subgroup differences in composite math scores by cerebral infarct status suggests that there was a statistically significant subgroup effect (*p* < 0.001), meaning that cerebral infarct status significantly affects composite math scores. However, there was substantial unexplained heterogeneity between the studies within each of the subgroups (no CVA: *I*^2^ = 33.4%, silent infarct: *I*^2^ = 34%, and stroke *I*^2^ = 75.2%). The mean math score was 76.2 (95% CI: 70.7–81.7) in the stroke group, 85.5 (95% CI: 80.8–90.2) in the silent infarct group, and 90 (95% CI: 85.9, 94.2) in the group without history of a CVA.

### Publication Bias

The regression test (37) was used to test for publication bias via funnel plot asymmetry. Egger's Tests using two-tailed criterion at *p*-values < 0.05 were flagged for potential funnel plot bias and include: percentage retained in the SCD population (*p* < 0.01) and the composite reading scores among controls (*p* = 0.03).

As a sensitivity analysis, we used a trim and fill data augmentation technique (39–41) to estimate how many studies would need to be included above or below the meta-analytic mean to make the funnel plot symmetrical, and to estimate how the hypothetical missing studies might affect the estimated grade retention estimate. The trim and fill yielded an estimate of the need to add 4 (SE = 2.7) studies on the left side (Supplementary Figure 1), and the resulting estimated rate of grade retention was 23% (95% CI: 17–30%).

## Discussion

Students with SCD demonstrate deficits in academic achievement across domains compared to normative expectations and healthy/sibling controls. This achievement gap appears to worsen as students with SCD age and demonstrate slowed academic growth. Students with SCI or stroke displayed greater achievement deficits than their peers without history of CVA. Despite frequently receiving formal academic supports, students with SCD are retained at an alarming rate.

This is the first meta-analysis examining academic performance in children with SCD in the United States. Compared to normative expectations, students with SCD demonstrated substantial deficits in reading, arithmetic, and spelling. These deficits generally did not vary across academic domains, with consistent medium to large effects. A large portion of the variance in academic achievement was accounted for by intellectual functioning, particularly reading skills. This is consistent with findings in the general population, showing a strong correlation between intellectual functioning and academic knowledge and attainment (42, 43). Slowed academic development is likely due in part to accumulated micro-infarcts, chronic hypoxemia, and repeated tissue ischemia as well as the cumulative impact of school absences. Academic deficits were reduced when compared to healthy and/or sibling controls, likely due to the contribution of sociodemographic factors. Most studies assessing academic achievement in SCD only reported a single subtest for each academic domain or a composite index, precluding more granular analysis of academic weaknesses. Only two of the included studies (11, 44) reported measures of academic fluency. In both studies, fluency measures were a relative academic weakness, consistent with processing speed deficits observed in SCD (45).

The implementation of school services differed notably across studies. Provision of IEPs and 504 Plans ranged from 14 to 74% and 4 to 16%, respectively. As expected, students with SCD were over twice as likely to receive an IEP than a national sample of African American students. These findings are consistent with other chronic medical conditions of childhood, such as congenital heart disease (46). Due to a limited number of studies, we were unable to examine if cognitive and/or academic performance were associated with provision of school services. Among a national sample of students diagnosed with attention deficit/hyperactivity disorder, provision of an IEP was associated with disease severity, diagnosis of a developmental delay and/or neurodevelopmental disorder, academic, and cognitive performance (47). In contrast, implementation of a Section 504 Plan was primarily associated with sociodemographic factors, such as primary language used in the home and type of health care coverage (47). Ghafuri et al. (48) observed that patients with SCD were more likely to receive school services following a neuropsychological evaluation. Yet, it is unclear if the results of the assessment resulted in improved access rather than advocacy by the providers. Conclusions drawn regarding school services are limited due to differing descriptions of special education services, and many studies did not differentiate between receiving a Section 504 Plan vs. an IEP (see Supplementary Table 3). Surprisingly, rates of services through Section 504 were much lower than those reported for an IEP. The definition of a disability under Section 504 is much broader than in an IEP and often includes children with no observed learning/cognitive deficits. Many families with limited resources may be unaware of accommodations provided through Section 504 (47) and would benefit from school advocacy. At a minimum, most children with SCD, regardless of cognitive/academic deficits, should qualify for accommodations under Section 504 to address medical concerns (e.g., bathroom breaks, excused absences for medical visits). For students with learning difficulties, accommodations and services should be informed through a more comprehensive evaluation done through the school and/or pediatric neuropsychologist.

On average, 27% of students with SCD were retained in at least one grade. These findings are concerning given the research on the long-term effects of grade retention demonstrating no benefits on academic attainment but negative effects on psychosocial outcomes (49). Students who are retained in grade are 50% more likely to drop out of high school (50) and their odds of attending college are cut in half, even after accounting for academic achievement, race, and socioeconomic status (51). King et al. (1) conducted the largest (*n* = 536) study assessing correlates of grade retention in students with SCD. They found that older age, male sex, and lower household income significantly increased risk of grade retention. Medical factors such as silent cerebral infarcts or frequency of pain episodes were not associated with grade retention. Because most studies did not report correlates of grade retention, we were unable conduct a meta-regression evaluating predictors of grade retention. It is recommended that medical and behavioral health providers advocate *against* retention for most students with SCD, instead recommending school services through an IEP or 504 Plan based on need.

Pooled effect sizes were smaller across academic achievement and attainment outcomes when compared to healthy controls relative to national normative data. These differences are likely accounted for by sociodemographic factors, as students with SCD tend to live in lower socioeconomic neighborhoods (3). Lower SES is associated with reduced academic achievement and attainment independent of disease status (52). A few of the included studies reported relationships between SES and academic achievement (19, 23, 53) or grade retention (1), but we were unable to calculate any pooled values due to the lack of standardization in the SES indices.

Poor academic attainment and grade retention have significant consequences. In the general population, reduced academic attainment is associated with poorer health literacy (54) and increased mortality (55), while grade retention is related to poorer psychosocial adjustment (56), delays in entering the workforce (57), and reduced salaries (58). Among patients with SCD, those without a high school education visit the emergency department three times as frequently as patients with post-secondary education (5). This association between educational attainment and hospital visits persists even after controlling for sociodemographic and disease factors (5). The academic attainment of patients with SCD has social and health consequences that go beyond the classroom environment. Further research is needed to evaluate the breadth of these academic consequences and potential intervention targets.

Individualized academic intervention and broader policy changes are needed to address the significant academic needs of students with SCD. Several institutions utilize academic liaisons to support patients with SCD from early childhood through adolescence. These liaisons typically work closely with neuropsychologists to screen patients for learning difficulties (59). If academic deficits and/or cognitive delays are identified, the academic liaisons can advocate for patients within the school environment and attend school meetings (e.g., IEP meetings). Further, liaisons can provide psychoeducation to families about their rights within the school environment and availability of academic resources, such as Section 504. Beyond services provided within the school environment, targeted academic interventions are needed. A majority of SCD patients lack academic readiness skills in preschool (60), and these early skills are the strongest predictor of later academic attainment (61, 62). Patients with SCD would benefit from increased access to early intervention, preschool services, and targeted academic readiness interventions.

Several study limitations exist. Due to variability in academic achievement measures across studies, we could not analyze specific areas of academic weakness beyond broad categories of mathematics, reading, and spelling. Future research should examine more specific academic outcomes (e.g., calculation, phonological processing, fluency). Moderation analyses were limited to age and intellectual functioning. Additional factors known to influence academic performance, such as socioeconomic status, could not be analyzed due to inconsistent reporting across studies. Factors which may affect academic performance also include missed school days due to outpatient appointments, illness and hospitalizations, as well as accrual of strokes or silent infarcts. Only a small number of studies reported academic achievement outcomes based on cerebral infarct status. From the available studies, it appears that there is a clear gradient of performance based on infarct severity. Further research is needed to understand the functional consequences of these cerebral vascular injuries and implications for academic intervention. There is also a need to understand the influence of infarct location and size on academic performance.

To conclude, the present meta-analysis clearly demonstrates the negative effects of SCD on academic achievement and attainment. Consistent with cognitive deficits, the academic achievement gap in math and reading appears to worsen as students with SCD age. A substantial portion of academic achievement is determined by levels of cognitive performance. Further research is needed to understand this relationship, and the specific cognitive domains that influence academic functioning in SCD. Despite well-documented academic deficits, most patients with SCD do not receive any form of academic services. Students with SCD would benefit from increased school advocacy and access to academic intervention services.

## Data Availability Statement

The original contributions presented in the study are included in the article/Supplementary Material, further inquiries can be directed to the corresponding author/s.

## Author Contributions

AH, JH, and LO contributed to the conception and design of the study. AH, LH, CS, and LO collected the data. JG and GK performed the statistical analyses. AH wrote the first draft of the manuscript. All authors contributed to manuscript revision, read, and approved the submitted version.

## Funding

JH received funding from U01HL133996 during the conduct of this study. AK received funding from R01HL129241, K24HL148305, K12HL137942, U01HL143477, and 5U01HL133994 during the time of his study. This research was supported by the American Lebanese Syrian Associated Charities (ALSAC).

## Conflict of Interest

AK and JH receive research funding from Global Blood Therapeutics. JH receives consultancy fees from Global Blood Therapeutics, Vindico Medical Education, UpToDate and bluebird bio. The remaining authors declare that the research was conducted in the absence of any commercial or financial relationships that could be construed as a potential conflict of interest.

## Publisher's Note

All claims expressed in this article are solely those of the authors and do not necessarily represent those of their affiliated organizations, or those of the publisher, the editors and the reviewers. Any product that may be evaluated in this article, or claim that may be made by its manufacturer, is not guaranteed or endorsed by the publisher.
