# Corrigendum: Academic performance of children with sickle cell disease in the United States: a meta-analysis

**DOI:** 10.3389/fneur.2023.1281630

**Published:** 2023-12-01

**Authors:** Andrew M. Heitzer, Latacha Hamilton, Claire Stafford, Jeffrey Gossett, Lara Ouellette, Ana Trpchevska, Allison A. King, Guolian Kang, Jane S. Hankins

**Affiliations:** ^1^Psychology, St. Jude Children's Research Hospital, Memphis, TN, United States; ^2^School Program, St. Jude Children's Research Hospital, Memphis, TN, United States; ^3^Psychology, Nova Southeastern College of Psychology, Fort Lauderdale, FL, United States; ^4^Biostatistics, St. Jude Children's Research Hospital, Memphis, TN, United States; ^5^Health Sciences Resource Center, Texas Medical Center Library, Houston, TX, United States; ^6^Program in Occupational Therapy and Departments of Pediatrics and Medicine, Washington University, St. Louis, MO, United States; ^7^Hematology, St. Jude Children's Research Hospital, Memphis, TN, United States

**Keywords:** sickle cell, anemia, academic performance, neurocognitive, education, stroke, silent infarct, grade retention

In the published article, there was an error. One of our analyses examined how rates of grade retention in children with sickle cell disease compared to national averages for Black/African American students. To make this comparison, we pulled data from the U.S. Department of Commerce, Census Bureau, Current Population Survey average retention rates for Black students (K-12) from 1994 to 2016 (https://nces.ed.gov/programs/digest/d17/tables/dt17_225.90.asp?referer=raceindicators). The average came out to ~3.8%. Since publishing this article, we have learned that these rates measure how likely a child is to be retained within a grade each year (only a single year time period). Whereas the values pulled for the systematic review were based on being retained at any time across grades. Therefore, this comparison may be overestimating how many children with sickle cell disease are retained compared to the national average.

As a result of this **Figure 4** needs to be removed from the article and the corrected Table 2 and its caption is below.

**Table 2:** Provision of special education services and rates of grade retention in sickle cell disease compared to rates for African Americans nationally.

**Outcome**	***k***	***n***	**Percentage (95% CI)**	**OR (95% CI)**	***p*-value**
Special education	19	1,669	38% (29%, 47%)	**–**	**–**
504 plan	5	808	10% (6%, 15%)	**–**	**–**
Individualized Education Plan (IEP)	10	1,210	32% (22%, 41%)	2.4 (1.5, 3.7)	**< 0.001**
Retention	16	1,803	27% (22%, 33%)		

*k*, number of unique studies included in analyses; *n*, total sample size from all included studies; CI, confidence interval; OR, odds ratio for comparison to normative data. Normative values for IEP services (IDEA Part B) were based on the average percentage of Black students (3–21 years) receiving services through IDEA Part B from 2000 to 2019. IEP normative value of 15.6% based on average counts per year from 2000 to 2019: (1,261,827/8,068,662). *p*-value < 0.05 was in bold.

A correction has been made to the Abstract, Design. This sentence previously stated:

“Prevalence of grade retention and special education services were compared to national (United States) estimates for Black students.”

The corrected sentence appears below:

“Prevalence of special education services was compared to national (United States) estimates for Black students.”

A correction has been made to the Abstract, Results. This sentence previously stated:

“Compared to unaffected sibling and/or healthy controls (*k* = 8, *n* = 508), reading and math scores were 0.40 (*p* = 0.017) and 0.36 (*p* = 0.033) SD below expectations. Grade retention was approximately 10 times higher in students with sickle cell disease than Black students nationally.”

The corrected sentence appears below:

“Compared to unaffected sibling and/or healthy controls (*k* = 8, *n* = 508), reading and math scores were 0.40 (*p* = 0.017) and 0.36 (*p* = 0.033) SD below expectations.”

A correction has been made to Methods, Data Extraction and Coding, paragraph 2. This sentence previously stated:

“Normative data for IEP services (IDEA Part B) and grade retention were extracted from U.S. national datasets. Grade retention normative values were based on the U.S. Department of Commerce, Census Bureau, Current Population Survey average retention rates for Black students (K-12) from 1994 to 2016 (32).”

The corrected sentence appears below:

“Normative data for IEP services (IDEA Part B) and grade retention were extracted from U.S. national datasets.”

A correction has been made to Methods, Statistical Analyses, paragraph 4.This paragraph previously stated:

“Pooled estimates of the proportions using services of Special education, 504, and IEP services and grade retention were estimated using random effects models. Heterogeneity of studies was also evaluated. For IEP's and grade retention, normative data were available, and comparisons were made via odds-ratios. Normative values for IEP services (IDEA Part B) were based on the average percentage of African-American students (3–21 years) receiving services through IDEA Part B from 2000 to 2019. Retention normative value of 3.8% based on average counts per year from 1994 to 2016: (285,749/752,462). IEP normative value of 15.6% based on average counts per year from 2000 to 2019: (1,261,827/8,068,662).”

The corrected paragraph appears below:

“Pooled estimates of the proportions using services of Special education, 504, and IEP services and grade retention were estimated using random effects models. Heterogeneity of studies was also evaluated. For IEP's, normative data were available, and comparisons were made via odds-ratios. Normative values for IEP services (IDEA Part B) were based on the average percentage of African-American students (3–21 years) receiving services through IDEA Part B from 2000 to 2019.”

A correction has been made to Results, IEP Support and Grade Retention, Paragraph 2. This paragraph previously stated:

“Based on 16 studies including rates of grade retention, the pooled estimate was 27% (22, 33%). The estimated average odds ratio based on the random-effects model was 9.7 (95% CI: 7.4–12.7), *p* < 0.001 (Table 2 and Figure 4). Thus, children with SCD were almost 10 times more likely to be grade-retained compared with African American children nationally.”

The corrected paragraph appears below:

“Based on 16 studies including rates of grade retention, the pooled estimate was 27% (22, 33%).”

A correction has been made to Discussion, paragraph 4. This sentence previously stated:

“The odds of grade retention for students with SCD were ~10 times greater than African American students nationally. On average, 27% of students with SCD were retained in at least one grade.”

The corrected sentence appears below:

“On average, 27% of students with SCD were retained in at least one grade.”

The authors apologize for these errors and state that this does not change the scientific conclusions of the article in any way. The original article has been updated.

