# Supplementary figures and images for: Academic Performance of Children With Sickle Cell Disease in the United States: A Meta-Analysis
Source: Front Neurol. 2021 Dec 13;12:786065. doi: 10.3389/fneur.2021.786065 (PMC8711768; doi:10.3389/fneur.2021.786065)

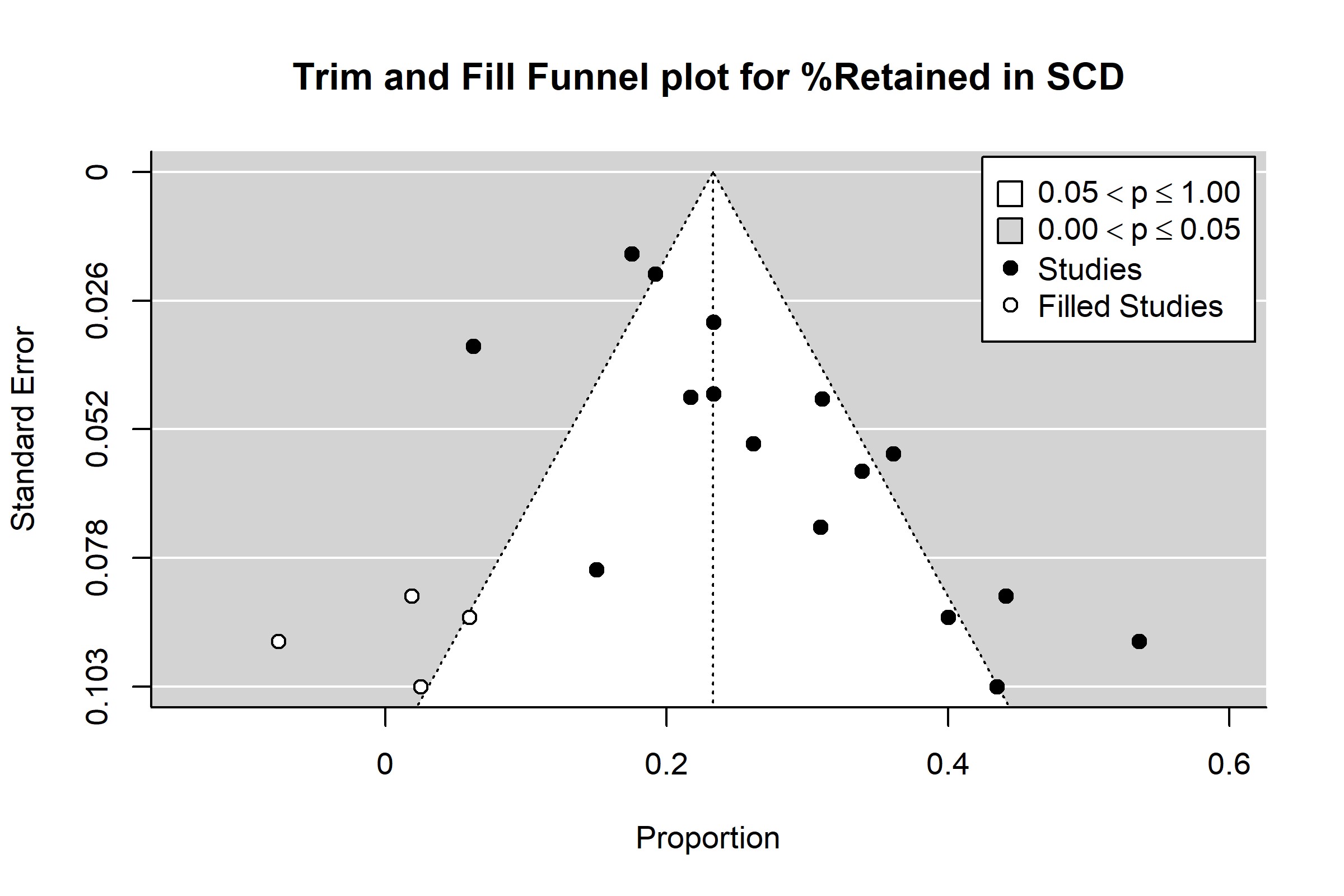

Supplement: Supplementary file 1 [file Image_1.JPEG]

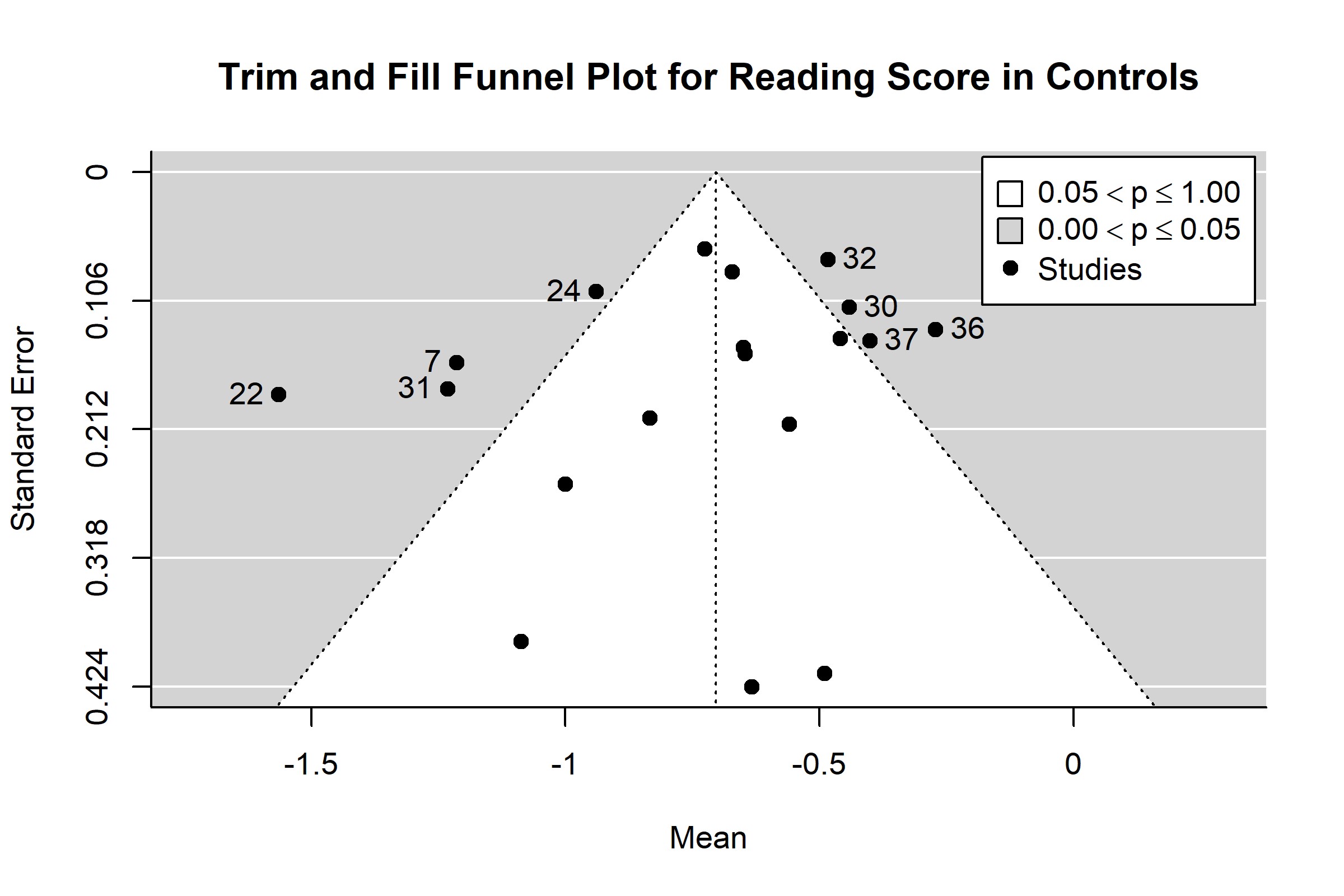

Supplement: Supplementary file 2 [file Image_2.JPEG]
