# Supplementary material for: Academic Performance of Children With Sickle Cell Disease in the United States: A Meta-Analysis
Source: Front Neurol. 2021 Dec 13;12:786065. doi: 10.3389/fneur.2021.786065 (PMC8711768; doi:10.3389/fneur.2021.786065)
Supplement: Supplementary file 3 [file Data_Sheet_1.docx]

Supplemental Table 1. Original Search Strategy Developed for Medline OVID

| 1. exp Anemia, Sickle Cell/ |
| --- |
| 1. ("sickl* cell*" or "sickel* cell*" or sickling* or scd).ti,ab,kw. |
| 1. ("hemoglobin S" or "hemo-globin S").ti,ab,kw. |
| 1. 1 or 2 or 3 |
| 1. exp Educational Measurement/ |
| 1. ((educat* or academic* or cognit* or school*) adj3 (measur* or performance* or score* or test* or achiev*)).ti,ab,kw. |
| 1. ((read* or writ* or math* or arithmetic*) adj3 (measur* or performance* or score* or test* or achiev*)).ti,ab,kw. |
| 1. exp Neuropsychology/ |
| 1. (neuropsych* or "neuro-psych*").ti,ab,kw. |
| 1. Cognitive Dysfunction/ |
| 1. ((cognitiv* or nerocognitiv* or neuro-cognitiv*) adj3 (develop* or function* or dysfunction* or defect*)).ti,ab,kw. |
| 1. 5 or 6 or 7 or 8 or 9 or 10 or 11 |
| 1. exp adolescent/ or exp child/ |
| 1. (child* or children* or boy or boys or girl* or youth* or pediatric* or paediatric* or kid or kids or "school-age*" or juvenile* or preteen* or tween*).ti,ab,kw. |
| 1. (teen* or adolescen* or minor or minors or pubescen* or "under-age*" or underage* or "high school*").ti,ab,kw. |
| 1. 13 or 14 or 15 |
| 1. 4 and 12 and 16 |
| A medical librarian (L.O.) developed the primary search in Medline, with three main concepts: (1) sickle cell, (2) academic performance and achievement, and (3) children. Each concept was developed using both controlled and natural languages. MeSH terms were identified, and keywords were gathered along with various synonyms. The keywords were searched using the title, abstract, and keyword fields within the Medline OVID database before being translated to other databases. |

| Supplemental Table 2. Sample characteristics, study quality, and performance measures for included studies | | | | | | | | |
| --- | --- | --- | --- | --- | --- | --- | --- | --- |
| **Year** | **First Author (Overlapping Sample)** | **SCD Sample Size** | **Control Sample Size** | **Age, years**  **Mean (Range)** | **SCD Genotype** | **Study Quality^a^** | **IQ Measure(s)** | **Academic Measure(s)** |
| 1988 | Fowler (64) | 28 | 28 | 11.3 (6-17) | HbSS | 4 | -- | WRAT-R |
| 1989 | Swift (65) | 21 | 21 | 11.5 (7-16) | HbSS | 5 | WISC-R | WJ |
| 1991 | Wasserman (66) | 43 | 30 | 11.5 (8-16) | HbSS, HbSb0 thal, HbSb+ thal, HbSC | 4 | WISC-R | WRAT |
| 1993 | Brown (A) (54) | 26 | 18 | 9.4 (7-13) | HbSS, HbSb0 thal, HbSC | 4 | KABC | KABC |
| 1994 | Barbarin (67) | 327 | -- | -- (4-17) | -- | 3 | -- | -- |
| 1995 | Eaton (68) | 21 | -- | -- (8-18) | HbSS, HbSb0 thal, HbSb+ thal, HbSC | 3 | -- | WRAT-R |
| 1996 | Armstrong (B) (69) | 194 | -- | 8.4 (6-12) | HbSSS | 5 | WISC-R | WJ-R |
| 1997 | Richard (70) | 42 | 26 | 7.9 (7-11) | -- | 4 | -- | CAT |
| 1998 | Devine (A) | 74 | -- | 9.8 (5-17) | HbSS, HbSb0 thal, HbSb+ thal, HbSC | 4 | WISC-3 | WJ-R |
| 1999 | Thompson (B) (22) | 289 | -- | 8.3 (5-15) | HbSS, HbSC | 4 | WISC-R | WJ-R |
| 2000 | Brown (A) (71) | 63 | -- | 9.8 (6-17) | HbSS, HbSC | 3 | WISC-3 | WJ-R |
| 2001 | Ievers-Landis (72) | 37 | -- | 8.7 (5-13) | HbSS, HbSb0 thal, HbSC | 5 | -- | -- |
| 2001 | Noll (C) (73) | 31 | 31 | 11.9 (9-16) | HbSS, HbSb0 thal, HbSb+ thal, HbSC | 6 | WISC-R | WRAT-R |
| 2001 | Wang (B) (74) | 247 | -- | 11.9 (6-18) | HbSS | 4 | WISC-R, WISC-3 | WJ-R |
| 2002 | Nabors (75) | 26 | 13 | 10.5 (6-13) | HbSS | 4 | -- | WRAT-R |
| 2003 | Kral (D) (76) | 60 | -- | 10.1 (6-16) | HbSS | 5 | WASI | WJ-R |
| 2004 | Grueneich (C) (77) | 31 | -- | 11.8 (9-16) | HbSS, HbSb0 thal, HbSb+ thal, HbSC | 4 | -- | WRAT-R |
| 2004 | Kral (D) (78) | 62 | -- | 10.1 (6-16) | HbSS | 6 | WASI | -- |
| 2004 | Schatz (E) (23) | 50 | 36 | 11.6 (7-17) | HbSS, HbSb+ thal, HbSC | 4 | WJ-R | WRAT-3 |
| 2005 | Peterson (79) | 72 | -- | 10.9 (5-17) | HbSS, HbSb0 thal, HbSb+ thal, HbSC | 3 | WISC-3, WAIS-3, SB-4 | WRAT-3 |
| 2005 | Woodard (80) | 9 | -- | 11.5 (8-17) | HbSS, HbSb0 thal | 3 | WISC-3, WAIS-3 | WRAT-3 |
| 2006 | King (81) | 23 | -- | 12 (5-19) | -- | 5 | -- | -- |
| 2006 | Kral (D) (82) | 27 | -- | 10.8 (6-16) | HbSS | 5 | WASI | WJ-R |
| 2006 | White (83) | 65 | -- | 11.5 (6-16) | -- | 4 | WASI | WIAT |
| 2008 | Gold (84) | 65 | -- | 13.0 (7-17) | HbSS, HbSb0 thal, HbSC | 3 | -- | WJ-R |
| 2009 | Schatz (E) (85) | 50 | 36 | 6.5 (5-7) | HbSS, HbSb0 thal, HbSb+ thal, HbSC | 4 | -- | WJ-3 |
| 2010 | Puffer (E) (86) | 64 | 81 | 6.4 (4-8) | HbSS, HbSb0 thal, HbSb+ thal, HbSC | 6 | -- | WJ-3 |
| 2011 | Mayes (87) | 83 | -- | 10.9 (4-18) | HbSS, HbSb0 thal, HbSb+ thal, HbSC | 3 | -- | -- |
| 2012 | Berg (F) (88) | 22 | 22 | 10.2 (8-12) | HbSS, HbSb0 thal | 3 | -- | -- |
| 2012 | Daly (89) | 34 | -- | 11.5 (6-16) | HbSS, HbSb0 thal, HbSb+ thal, HbSC | 4 | WASI | -- |
| 2013 | Epping (90) | 197 | -- | 11.5 (5-18) | HbSS, HbSb0 thal, HbSb+ thal, HbSC | 4 | -- | -- |
| 2013 | Smith (19) | 82 | -- | 8.4 (6 to 12) | HbSS, HbSb0 thal, HbSb+ thal, HbSC | 5 | WASI | WJ-3 |
| 2014 | King (1) | 536 | -- | 9.4 (5 to 15) | HbSS, HbSb0 thal | 5 | -- | -- |
| 2014 | Ladd (91) | 370 | -- | 10.6 (6 to 16) | HbSS, HbSb0 thal, HbSb+ thal, HbSC | 4 | -- | WJ-R |
| 2015 | Crosby (20) | 30 | -- | 16.0 (12 to 20) | HbSS, HbSb0 thal, HbSb+ thal, HbSC | 5 | -- | -- |
| 2017 | Yarboi (F) (11) | 65 | -- | 11.2 (6-16) | HbSS, HbSb0 thal, HbSb+ thal, HbSC | 5 | WASI | WJ-3 |
| 2018 | Prussien (G) (92) | 44 | -- | 9.3 (6-16) | HbSS, HbSb0 thal, HbSb+ thal, HbSC | 5 | -- | -- |
| 2018 | Schatz (E) (93) | 90 | -- | 6.5 (5-7) | HbSS, HbSb0 thal, HbSb+ thal, HbSC | 4 | -- | WJ-3 |
| 2019 | Bills (E) (94) | 65 | 59 | 6.3 (4-8) | HbSS, HbSb0 thal, HbSb+ thal, HbSC | 3 | -- | WJ-3 |
| 2019 | Connolly (95) | 89 | -- | 10.4 (7-16) | HbSS, HbSb0 thal, HbSb+ thal, HbSC | 4 | WISC-5 | WJ-3 |
| 2019 | Ghafuri (49) | 42 | -- | 11.0 (3-18) | HbSS, HbSb0 thal, HbSb+ thal, HbSC | 4 | WAIS-IV, WISC-IV, WPPSI-3, KABC-2 | -- |
| 2019 | Yarboi (G) (96) | 65 | -- | 9.3 (6-16) | HbSS, HbSb0 thal, HbSb+ thal, HbSC | 4 | WASI-2 | WRAT-4 |
| 2020 | Karkoska (97) | 20 | -- | 8.2 (6-9) | HbSS, HbSb0 thal, HbSb+ thal, HbSC | 5 | -- | -- |
| 2020 | Partanen (45) | 103 | -- | 16.8 (15-18) | HbSS, HbSb0 thal, HbSb+ thal, HbSC | 5 | WASI-2 | WJ-3 |
| --, information not available or not relevant; SCD, sickle cell disease; WRAT, Wide Range Achievement Test; WISC, Wechsler Intelligence Scale for Children; KABC, Kaufman Assessment Battery for Children; CAT, California Achievement Test; SRA, Science Research Associates Achievement Series; ITBS, Iowa Test of Basic Skills; WJ, Woodcock Johnson; WASI, Wechsler Abbreviated Scale of Intelligence; WAIS, Wechsler Adult Intelligence Scale; SB, Stanford Binet Intelligence Scale, WPPSI, Wechsler Preschool and Primary Scale of Intelligence.  ^a^ A version of the National Institutes of Health Quality Assessment Tool for Observational Cohort and Cross-Sectional Studies adapted by Prussien and colleagues was utilized. Studies received one point for each criterion met, for a total score of 0 to 6. | | | | | | | | |

| Supplemental Table 3. Description of educational services across studies | | |
| --- | --- | --- |
| **Year** | **First Author** | **Description of services provided** |
| 1988 | Fowler | Special services received |
| 1991 | Wasserman | Special education placement |
| 1994 | Nettles | Special education |
| 1998 | Devine | Special education placement: receiving special education services |
| 2000 | Brown | Special education placement |
| 2001 | Ievers-Landis | Receiving special education |
| 2003 | Kral | Special education placement |
| 2004 | Kral | Special education placement: receipt of special education services either in the regular education classroom (504) or in an alternative classroom placement (e.g., resource classroom or self-contained classroom) |
| 2004 | Schatz | Needed academic services |
| 2005 | Peterson | Receiving special services or had an individualized education plan |
| 2006 | King | Presence of an individualized education plan |
| 2006 | Kral | Special education placement: receipt of special education services either in the regular classroom (e.g., 504) or in an alternative classroom placement (e.g., resource classroom or self-contained classroom) |
| 2011 | Mayes | Currently has an individualized education plan or currently has a 504 plan |
| 2012 | Daly | Special education placement |
| 2013 | Epping | Special education services: receipt of special education services through an individualized education program either in the regular classroom or special education classroom at any time in the child’s life |
| 2013 | Smith | Individualized education or 504 plan |
| 2014 | King | Presence of an individualized education plan |
| 2015 | Crosby | Special education placement (504 or individualized education plan) |
| 2017 | Yarboi | Individualized education plan, 504 Plan |
| 2018 | Prussien | Special education placement (504 or individualized education plan) |
| 2018 | Schatz | Individualized education plan |
| 2019 | Ghafuri | Special education services (504 or individualized education plan) |
| 2019 | Yarboi | Receiving special education services at school (e.g. individualized education plan, 504 plan) |
| 2020 | Karkoska | Presence of an individualized education plan |
| 2020 | Partanen | Received supports at school |

**Supplemental Figure 1.** Trim and fill funnel plot for rates of retention among students with sickle cell disease. Egger’s test was flagged for potential funnel plot bias. The trim and fill yielded an estimate of the need to add 4 (SE=2.7) studies on the left side, and the resulting estimated rate of grade retention was 23% (95% CI: 17% to 30%).

**Supplemental Figure 2.** Trim and fill funnel plot for reading performance among controls. Egger’s test was flagged for potential funnel plot bias.
